# Loss of tissue-type plasminogen activator causes multiple developmental anomalies

**DOI:** 10.1093/braincomms/fcae408

**Published:** 2024-11-16

**Authors:** Kevin Uguen, Tanja Frey, Osama Muthaffar, Jean-Claude Décarie, Najim Ameziane, Sarah Boissel, Yalda Baradaran-Heravi, Anita Rauch, Gabriela Oprea, Aboulfazl Rad, Katharina Steindl, Jacques L Michaud

**Affiliations:** CHU Sainte-Justine Azrieli Research Centre, Montreal H3T 1C5, Canada; Department of Medical Genetics, CHRU Brest, Brest F 29200, France; Univ Brest, Inserm, EFS, UMR 1078, GGB, Brest F-29200, France; Institute of Medical Genetics, University of Zürich, Schlieren-Zurich 8952, Switzerland; Department of Pediatrics, Faculty of Medicine, King Abdulaziz University, Jeddah 21589, Saudi Arabia; Department of Medical Imaging, CHU Sainte-Justine, Montreal, Quebec H3T 1C5, Canada; Department of Radiology, Radio-Oncology and Nuclear Medicine, Université de Montréal, Montreal, Quebec H3C 3J7, Canada; Arcensus GmbH, Rostock 18119, Germany; CHU Sainte-Justine Azrieli Research Centre, Montreal H3T 1C5, Canada; Institute of Medical Genetics, University of Zürich, Schlieren-Zurich 8952, Switzerland; Institute of Medical Genetics, University of Zürich, Schlieren-Zurich 8952, Switzerland; University Children’s Hospital Zurich, Zurich 8032, Switzerland; University of Zurich Research Priority Program ITINERARE: Innovative Therapies in Rare Diseases, Zurich 8952, Switzerland; University of Zurich Research Priority Program AdaBD: Adaptive Brain Circuits in Development and Learning, Zurich 8952, Switzerland; Arcensus GmbH, Rostock 18119, Germany; Arcensus GmbH, Rostock 18119, Germany; Institute of Medical Genetics, University of Zürich, Schlieren-Zurich 8952, Switzerland; CHU Sainte-Justine Azrieli Research Centre, Montreal H3T 1C5, Canada; Department of Pediatrics, Université de Montréal, Montreal H3C 3J7, Canada; Department of Neurosciences, Université de Montréal, Montreal H3C 3J7, Canada

**Keywords:** hydrocephalus, Dandy–Walker malformation, *PLAT*, plasminogen, intellectual disability

## Abstract

Hydrocephalus and Dandy–Walker malformation are amongst the most common congenital brain anomalies. We identified three consanguineous families with both obstructive hydrocephalus and Dandy–Walker malformation. To understand the molecular basis of these anomalies, we conducted genome-wide sequencing in these families. We identified three homozygous truncating variants in the *PLAT* gene in the four affected family members. All of them showed tetraventricular hydrocephalus. In two individuals, a membrane at the inferior aspect of the fourth ventricle was likely the cause of their hydrocephalus. Three cases exhibited Dandy–Walker malformation, whereas the two oldest individuals displayed intellectual disability. *PLAT* encodes the tissue-type plasminogen activator, a serine protease whose main function is to cleave the proenzyme plasminogen to produce active plasmin. Interestingly, plasminogen deficiency has also been shown to cause obstructive hydrocephalus and Dandy–Walker malformation, suggesting that loss of *PLAT* causes these defects by disrupting plasmin production. In summary, we describe a recessive disorder characterized by obstructive hydrocephalus, Dandy–Walker malformation and intellectual disability in individuals with loss-of-function variants in *PLAT*. This discovery further strengthens the involvement of the plasminogen pathway in the pathogenesis of these developmental disorders.

## Teaser

Uguen *et al.* report four cases presenting with hydrocephalus and Dandy–Walker malformations harbouring homozygous loss-of-function variants in the *PLAT* gene. *PLAT* encodes the tissue plasminogen activator, and, based on the phenotype associated with plasminogen deficiency, authors propose that the disruption of the plasminogen pathway is responsible of brain malformations.

## Graphical Abstract

**Graphical Abstract:**
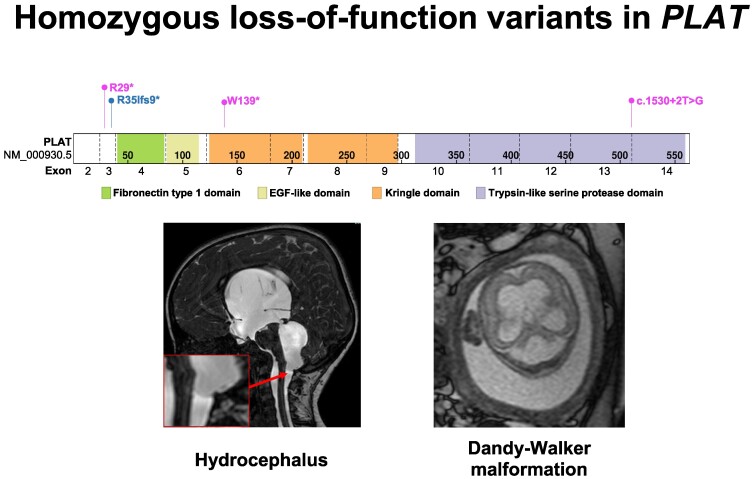

## Introduction

Congenital brain anomalies represent a major cause of neuro-developmental disabilities. Two of the most common anomalies amongst this group are hydrocephalus and Dandy–Walker malformation (DWM). Congenital hydrocephalus is characterized by an accumulation of CSF within the brain, detected prenatally or during infancy^1,2^. Classically, hydrocephalus is classified as obstructive or non-communicating when it is caused by an obstruction of CSF flow within the ventricles and as communicating when it is caused by CSF flow obstruction or decreased absorption in the subarachnoid spaces.^1,2^ DWM is defined by the presence of cerebellar vermis hypoplasia, dilatation of the fourth ventricle and enlarged posterior fossa with upward displacement of the tentorium.^3^ The molecular basis of congenital hydrocephalus and DWM remains poorly understood. Here, we describe homozygous truncating variants in the *PLAT* gene in four cases, from three consanguineous families, presenting with obstructive hydrocephalus and DWM.

## Materials and methods

This study was approved by the CHU Sainte-Justine’s research ethics board. Informed consent was obtained from all participants and data were de-identified. In Family 1, exome sequencing was performed in affected siblings, as previously described.^4^ In the other families, exome (Family 2) or genome (Family 3) sequencing was performed on a clinical basis. Description of the variants in *PLAT* is based on the National Center for Biotechnology Information reference sequence NM_000930.5, using complementary DNA numbering with position 1 corresponding to the A of the ATG translation initiation codon. Variants are also described at the chromosomal level (GRCh38) using the reference sequence NC_000008.11 and at the protein level using the reference sequence NP_000921.1 (Table 1).

**Table 1:** Summary of findings in individuals with variants in *PLAT*

Case	1.1	1.2	2.2	3.1	4.1	4.2
**Description**	This report	This report	This report	This report	Shamseldin *et al*.^10^	Shamseldin *et al*.^10^
**Sex**	M	F	M	F	M	M
**Age**	22 years	Foetus	8 years	14 months	12 h (deceased)	26 days (deceased)
**Consanguinity**	Yes	Yes	Yes	Yes	Yes	Yes
**Variants**						
** NM_000930.5**	c.85C > T	c.85C > T	c.1530 + 2T > G	c.416G > A	c.102_103del	c.102_103del
** NC_000008.11**	g.42191402G > A	g.42191402G > A	g.42178895G > A	g.42187521C > T	g.42191384_ 42191385del	g.42191384_ 42191385del
** NP_000921.1**	p.(Arg29*)	p.(Arg29*)	p.?	p.(Trp139*)	p.(Arg35Ilefs*9)	p.(Arg35Ilefs*9)
** Status**	Homozygous	Homozygous	Homozygous	Homozygous	Homozygous	Homozygous
**Clinical manifestations**						
** Hydrocephalus**	Yes	Yes	Yes	Yes	Yes	Yes
** Dandy–Walker malformation**	No	Yes	Yes	Yes	No	No
** Cognitive impairment**	Intellectual disability	NA	Intellectual disability	Global developmental delay	NA	NA
** Epilepsy**	No	NA	Yes (focal)	Yes (focal)	NR	NR
** Heart defects**	Yes	No	No	No	Yes	Yes
** Diaphragmatic hernia**	No	No	No	No	Yes	Yes
** Hypospadias**	Yes	NA	Yes	NA	NR	NR

NA, not applicable; NR, not reported.

## Results

We initially encountered two siblings (Family 1) with brain structural anomalies (Table 1). Their parents are first cousins. The proband (1.1) is a 22-year-old male who presented prenatally with heart anomalies, including atrial septal defect, interruption of the inferior vena cava with azygos continuation and dilatation of the ascending aorta. At 7 years of age, he underwent heart surgery to patch the septal defect and replace his ascending aorta. He also exhibited hypospadias, which was corrected surgically.

**Figure 1:**
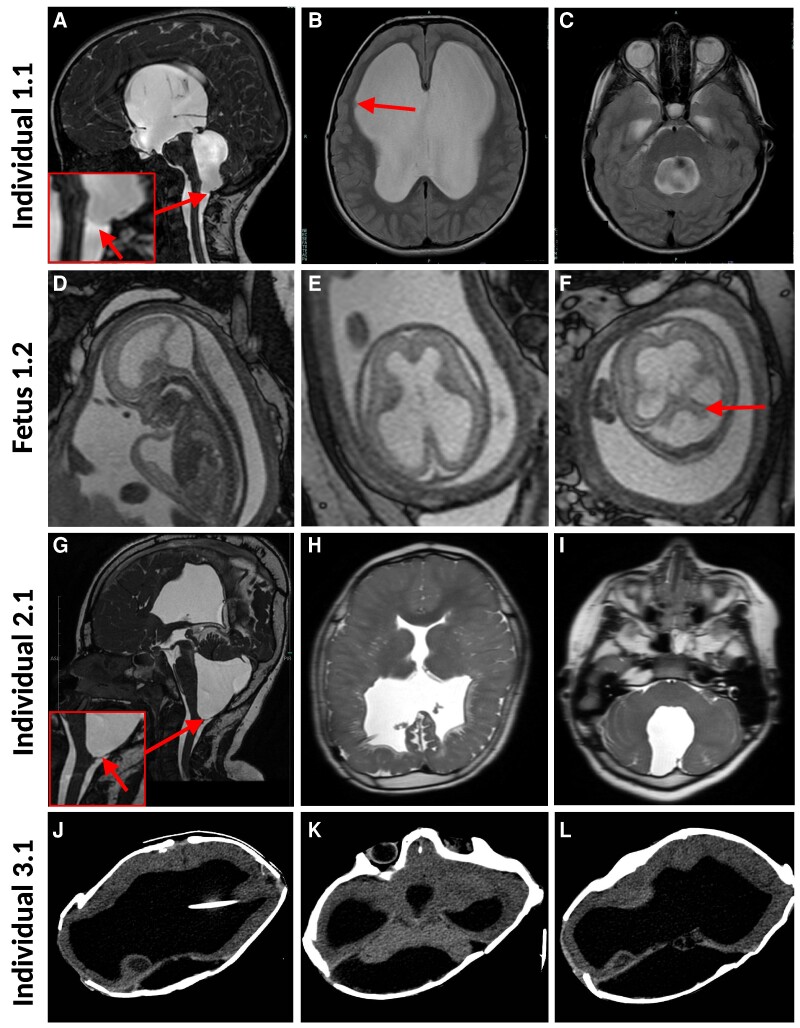
**Brain images of four individuals with a loss of *PLAT* function.** (**A–C**). MRI images from Individual 1.1 at 7 years of age. (**A**) Sagittal constructive interference in steady state (CISS) image. The arrow points to CSF flow artefacts (enlarged in the inset) suggesting the presence of a membrane (not visualized because of these artefacts) at the outlets of the fourth ventricle. (**B**) Axial T2 image showing significant enlargement of both lateral ventricles with frontal predominance and absent septum pellucidum and subependymal heterotopia (arrow). (**C**) Axial T2 image showing dilated 4th ventricle with turbulent cerebrospinal fluid. (**D–F**) Images from foetus 1.2 at 23 3/7 weeks of gestation. (**D**) Sagittal T2 image showing dilated fourth ventricle. (**E**). Axial T2 image showing dilatation of both lateral ventricles. **(F)**. Oblique coronal image showing hypoplastic cerebellum (arrow) and dilated fourth ventricle. **(G–I)**. MRI images from Individual 2.2 at 19 months of age. **(G)**. Sagittal 3D Fiesta showing a membrane at the central outlet of the fourth ventricle (arrow). **(H)**. Axial T2 reformat showing absent septum pellucidum, subependymal heterotopia and ventricular shunt with residual hydrocephalus. **(I)**. Enlargement of fourth ventricle with dysplastic cerebellar hemispheres. **(J–L)**. Axial CT-scan images from Individual 3.1 at 1 year of age, showing significant enlargement of both lateral ventricles (**J, K**) and the fourth ventricle **(L)**.

Increased head circumference was noted at around 21 months of age (53 cm, >+3 SD). Brain MRI performed at that age and several times over the following years revealed tetraventriculomegaly, an arachnoid cyst at the right pontocerebellar angle, subependymal heterotopia along the right frontal horn and a thin corpus callosum. MRIs also revealed the presence of a membrane at the inferior aspect of the fourth ventricle, which is likely the cause of the hydrocephalus in this individual (Fig. 1A–C). At 8 years of age, he presented with papilledema and progression of the ventriculomegaly, suggesting intracranial hypertension caused by his hydrocephalus. He was successfully treated by ventriculostomy of the third ventricle.

Individual 1.1 started to walk at 18 months of age. At 30 months of age, he could use two- to three-word sentences. He can currently communicate in full sentences. Psychological assessments showed mild intellectual disability and attention-deficit hyperactivity disorder. Chromosomal microarray was negative.

The mother subsequently became pregnant with a female foetus (1.2). Foetal ultrasound performed at 16 3/7 weeks of gestation revealed mild bilateral dilatation of the lateral ventricles (atria measured at 10.3 and 11.3 mm) and a cystic lesion in the posterior fossa. Another ultrasound performed at 19 0/7 weeks of gestation showed worsening of the ventriculomegaly (with the atria measured between 14 and 15 mm), dilatation of the third ventricle, and persistence of a cyst in the posterior fossa. Foetal MRI performed at 23 6/7 weeks of gestation revealed a further increase in the size of the lateral ventricles (atria measured at 20 mm), dilatation of the third (measured at 5 mm) and fourth ventricles, dilatation of the subarachnoid space of the posterior fossa with hypoplasia and upward displacement of the vermis, and hypoplasia of the brainstem (Fig. 1D–F). Therefore, the foetus exhibited tetraventriculomegaly, like her sibling, but also DWM. Foetal echocardiography performed at 20 1/7 weeks of gestation and karyotyping performed on amniotic fluid showed no anomalies. Parents opted to terminate the pregnancy at 25 weeks of gestation but declined an autopsy of the foetus.

**Figure 2:**
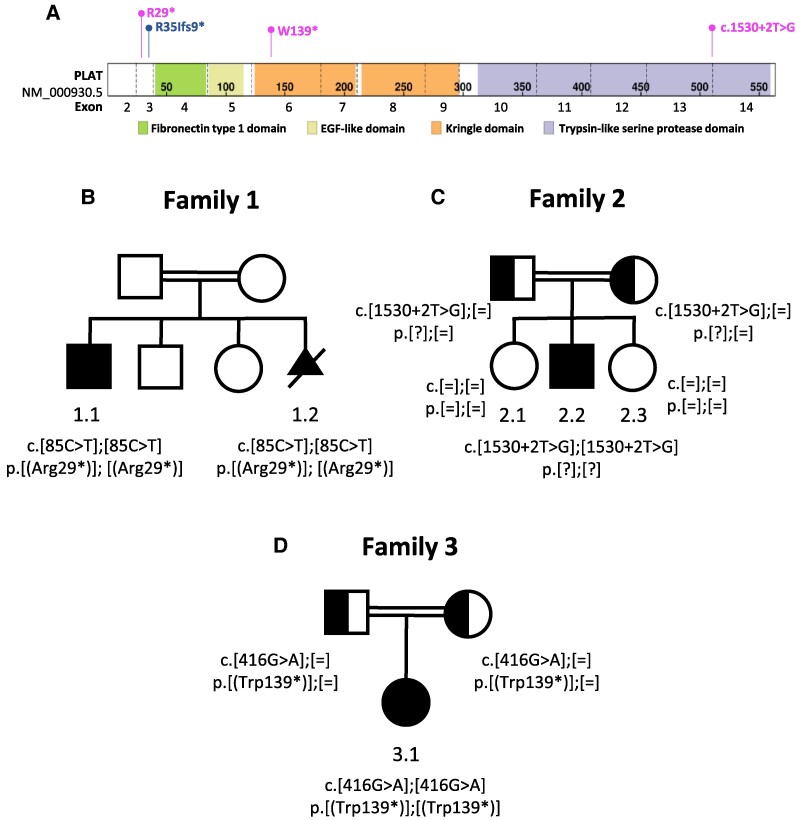
**Localization of truncating variants in *PLAT*. (A)**. Variants reported herein (R29*, W139* and c.1530+2T>G), and the previously reported variant (R35Ifs9*). Upper numbers represent the amino acid position, whereas lower numbers indicate the exons. Protein domains are represented using a colour code. Scheme created using Lollipop (https://proteinpaint.stjude.org/). **(B–D)** Pedigrees of the three families indicating the genotype at the *PLAT* locus in individuals who have undergone genetic testing. The black symbols represent the affected homozygous individuals. The half-filled symbols represent unaffected heterozygous individuals. The crossed-out triangle represents a termination of pregnancy.

To investigate the aetiology of the anomalies found in these siblings, we sequenced their exome. Analysis of each exome did not reveal any rare variants in OMIM genes that are associated with the phenotypes of the siblings. Because of the consanguinity of the parents, we next focused on rare homozygous variants. In total, we identified 82 such variants, including 14 variants shared by the siblings and 68 variants found in only one of them (Supplementary Table 1). Amongst these, one homozygous variant in the *PLAT* gene (c.85C > T, p.(Arg29*)), shared by the siblings, was of particular interest (Fig. 2A and B). This truncating variant was found 17 times in the gnomAD population database (V4.1.0) in the heterozygous state and is predicted to induce non-sense-mediated decay of the transcript. The *PLAT* gene encodes the tissue-type plasminogen activator (tPA; EC 3.4.21.68), a serine protease whose main function is to cleave the proenzyme plasminogen to produce active plasmin. Interestingly, individuals with plasminogen deficiency have been reported, like our siblings, to develop obstructive hydrocephalus and/or DWM (reviewed in Schuster *et al.*^5^). The presence of this variant in other family members could not be investigated because samples were not available. The other singleton or shared homozygous variants, were not likely to explain their conditions either because they were found in genes associated with unrelated disorders, they were predicted to be neutral by in silico prediction tools, or they involved a gene that is not expressed in the developing brain (Supplementary Table 1).

Using Matchmaker Exchange, we identified two other families with homozygous truncating variants in *PLAT* (Table 1). In Family 2, the proband (Individual 2.2) is an 8-year-old boy who was referred at the age of 6 months for macrocrania, hydrocephalus, DWM and mildly delayed first milestones. The boy was born at 36 weeks 4/7 of gestation with a birthweight of 3240 kg (−1 to 0 SD), a birth length of 48.5 cm (−1 to −2 SD) and a head circumference of 44.5 cm (>+3 SD) to consanguineous Turkish parents. DWM was detected prenatally at 21 weeks of gestation. Prenatal chromosomal microarray was normal. Postnatally, brain MRI revealed multiple subependymal heterotopias, absent septum pellucidum, parenchyma loss or hypoplasia of both parieto-occipital lobes with complete loss of the paramedian cortex on the right side, dysgenesis of the corpus callosum, dilation of the four ventricles and DWM (Fig. 1G–I). As was the case for Individual 1.1, MRIs also showed the presence of a membrane at the inferior aspect of the fourth ventricle (Fig. 1G). A ventriculoperitoneal shunt was inserted at 9 days of age. The boy exhibited hypospadias and cryptorchidism, which were subsequently corrected. He started to walk at 3 years and 1 month of age. At the age of 8 years, he could express himself in simple words in his native language. He is autonomous in some of the daily activities but is still not completely toilet trained. The individual shows mild facial anomalies including a broad forehead, deep-set eyes and low-set simplified ears. He suffers from focal epilepsy, which was partially controlled by anti-epileptic mono- or polytherapy, including levetiracetam, valproic acid, clobazam and lamotrigine.

Trio exome sequencing in Family 2 revealed the presence in Individual 2.2 of a homozygous variant in *PLAT* (c.1530 + 2T > G, p.?) affecting a canonical splice site (Fig. 2A and C). This variant is absent from gnomAD v2.1.1. SpliceAI^6^ and varSEAK (https://varseak.bio./) predict that the variant induces a loss of the donor site in intron 13 (Supplementary Table 2). varSEAK also predicts that loss of the site would lead to the skipping of the out-of-frame exon 13, removing Ser478, one of the three residues critical for tPA catalytic activity (Supplementary Table 2).^7^ The analysis did not reveal any rare variants in OMIM genes that are associated with the phenotype of the individual. Segregation analysis showed that the parents, but not the two unaffected siblings, carried the variant.

In Family 3, the proband is a girl (Individual 3.1) born to consanguineous parents from Saudi Arabia. She was delivered at 36 weeks of gestation by caesarean section because of hydrocephalus, which was diagnosed during the fourth month of pregnancy. Her head circumference at birth was 48 cm (> + 3 SD). A ventriculoperitoneal shunt was inserted during the first week after birth. A brain CT scan performed at 1 year of age showed severe dilation of the four ventricles and DWM (Fig. 1J–L). When last seen at 14 months of age, she was not alert to her surroundings and had poor eye contact. She had no neck support, could not roll over or reach out, and was not uttering any words. Additionally, she had focal seizures, which were well controlled with levetiracetam. At that age, weight was 5 kg (<−3 SD), length 60 cm (<−3 SD) and head circumference 48 cm (+2 SD). Neurological examination revealed mild hypotonia.

Genome sequencing performed in Individual 3.1 identified a homozygous non-sense variant in *PLAT* (c.416G > A, p.(Trp139*)) (Fig. 2A and D). This variant is absent from gnomAD v2.1.1 and v4.1.0 and is predicted to induce non-sense mediated decay of the transcript. Its presence in the proband and its segregation in the family was confirmed by Sanger sequencing. In addition, genome sequencing showed the presence of a homozygous pathogenic variant in *VPS13B* (NM_152564.5:c.1219C > T, p.(Gln407*)) in this individual. Bi-allelic loss-of-function variants in this gene cause Cohen syndrome (OMIM:216550), which is characterized by intellectual disability, microcephaly and retinopathy. However, this disorder is not associated with hydrocephalus and DWM. We postulate that this individual has two diagnoses, Cohen syndrome and a *PLAT*-related disorder, which explains her structural brain anomalies.

## Discussion

We found homozygous truncating variants in the *PLAT* gene in an aborted foetus and three individuals from consanguineous families. All of them showed tetraventricular hydrocephalus. In two individuals, we documented the presence of a membrane at the inferior aspect of the fourth ventricle, which is likely the cause of their hydrocephalus. In addition, two individuals and one foetus exhibited DWM, whereas the two oldest individuals exhibited mild intellectual disability.

The main function of *PLAT* product, tPA, is to cleave plasminogen to produce active plasmin, which is a potent serine protease with an extensive range of substrates, including fibrin, the chief constituent of thrombus. Plasminogen deficiency (OMIM:217090), due to bi-allelic variants in the *PLG* gene, results in deficits of fibrinolysis and the deposition of pseudomembranes on mucosal surfaces, causing mainly conjunctivitis and gingivitis but also inflammation of the respiratory, gastrointestinal and female genital tracts.^5,8^ It is noteworthy that congenital obstructive hydrocephalus and DWM have been found in 12% and 5% of individuals with plasminogen deficiency, respectively.^5^ The occurrence of hydrocephalus and DWM in individuals with loss of either *PLAT* or *PLG* strongly suggests that these brain anomalies are caused by the disruption of plasmin production (Supplementary Fig. 1).

The pathogenesis of congenital hydrocephalus in plasminogen deficiency appears to involve the obstruction of the cerebral ventricular system by the deposition of fibrin at the level of the aqueduct of Sylvius or drainage foramina of the fourth ventricle.^9-11^ Likewise, we postulate that the membranes observed at the lower aspect of the fourth ventricle in Individuals 1.1 and 2.1 correspond with the deposition of fibrin. As our two other cases showed dilatation of the four ventricles, it appears likely that their hydrocephalus is also caused by the deposition of fibrin, compromising the patency of the recesses of the fourth ventricle.

Interestingly, a homozygous frameshift variant in *PLAT*, located 17 base pairs downstream from the variant found in Family 1, has been previously reported in a consanguineous family with two siblings displaying hydranencephaly, cardiac defects (patent ductus arteriosus, ventricular septal defect) and diaphragmatic hernia (Table 1, Fig. 2).^12^ Western blot showed that the production of tPA was abolished in lymphoblastoid cells from one of the siblings.^12^ Hydranencephaly is a condition in which cerebral hemispheres are completely or almost completely absent and are replaced by cerebrospinal fluid.^13^ The marked macrocrania at birth in these siblings (head circumference at +3.8 and +10 SD) strongly suggest that their hydranencephaly resulted from severe obstructive hydrocephalus, which might have reduced the cerebral mantle to a thin membrane. All together, these observations further strengthen the association between loss of *PLAT* function and the development of obstructive hydrocephalus.

It remains unclear whether the pathogenesis of DWM associated with the loss of *PLAT* and *PLG* involves the same mechanism underlying the development of hydrocephalus. Recent studies suggest that defects of vascular development represent major factors for the pathogenesis of DWM.^14,15^ Interestingly, *PLAT* and *PLG* have been shown to play an important role in angiogenesis via a plasmin-dependent mechanism, raising the possibility that loss of their function might cause DWM by affecting the development of blood vessels (Supplementary Fig. 1).^16,17^

*PLAT* is expressed in neurons throughout the developing brain and it has been shown to play a role in multiple processes, including neuronal migration, axonal growth, synaptic plasticity and learning, through plasmin-dependent proteolytic, plasmin-independent proteolytic and/or non-proteolytic mechanisms (reviewed in Lee *et al.*^18^). Subependymal heterotopias, which are defects of neuronal migration, were observed in Individuals 1.1 and 2.2. Interestingly, subependymal heterotopias have also been reported in an individual with plasminogen deficiency.^19^ The observation of neuronal migration defects in patients with either tPA or plasminogen deficiency is consistent with the suggestion that these proteins influence neuronal migration by activating plasmin to break down cell adhesions or extracellular matrix.^18^ Cognitive deficits were observed in Individuals 1.1, 2.2 and 3.1. The more severe developmental phenotype observed in Individual 3.1 may be explained by the co-occurrence of Cohen syndrome. Developmental delay and intellectual disability have also been reported in some individuals with plasminogen deficiency.^10,20^ Further studies will be necessary to determine whether cognitive impairment is a cardinal feature of tPA and plasminogen deficiencies and whether it is secondary to hydrocephalus or is caused by the disruption of specific neuro-developmental processes involving plasmin-dependent or independent mechanisms.

In summary, we describe a disorder characterized by obstructive hydrocephalus, DWM and intellectual disability in individuals with loss-of-function variants in *PLAT*. This discovery further strengthens the involvement of the plasminogen pathway in the pathogenesis of these developmental disorders.

## Supplementary Material

fcae408_Supplementary_Data

## Data Availability

Data and materials individually are available upon request.
