# Supplementary material for: Loss of tissue-type plasminogen activator causes multiple developmental anomalies
Source: Brain Commun. 2024 Nov 16;6(6):fcae408. doi: 10.1093/braincomms/fcae408 (PMC11580217; doi:10.1093/braincomms/fcae408)
Supplement: fcae408_Supplementary_Data [file fcae408_supplementary_data.zip › Supplementary material.pdf]

Supplementary material

Supplementary Table 1. Homozygous variants detected in individual 1.1 and fetus 1.2

Supplementary Table 2. Splicing prediction of the variant from family 2

Supplementary Figure 1. Scheme illustrating the function of tPA and plasminogen and the impact of their disruption.

Splice predictor	Score	Predicted effect
**SpliceAI**	AG:0<br>AL:0<br>DG:0<br>**DL:0.99**	Loss of donor splice site.
**varSEAK**<br>**5' Donor Splice Site**	Score for reference:<br>+50.67%<br>**Score with variant: No GT**	Loss of function for authentic splice site.<br>Exon 13 skipping.

Supplementary Table 2. Splicing prediction of the variant from family 2. We used the following tools: spliceAI and varSEAK. AG = acceptor gain, AL = acceptor loss, DG = donor gain, DL = donor loss.

Supplementary Figure 1. Scheme illustrating the function of tPA and plasminogen and the impact of their disruption. (A) tPA cleaves plasminogen to produce active plasmin, which is required to break down fibrin and promote angiogenesis. (B) Loss of tPA and plasminogen function is predicted to impair fibrinolysis and angiogenesis. One possibility is that hydrocephalus and DDW in individuals with a loss of *PLAT* is caused by the disruption of fibrinolysis and angiogenesis, respectively.

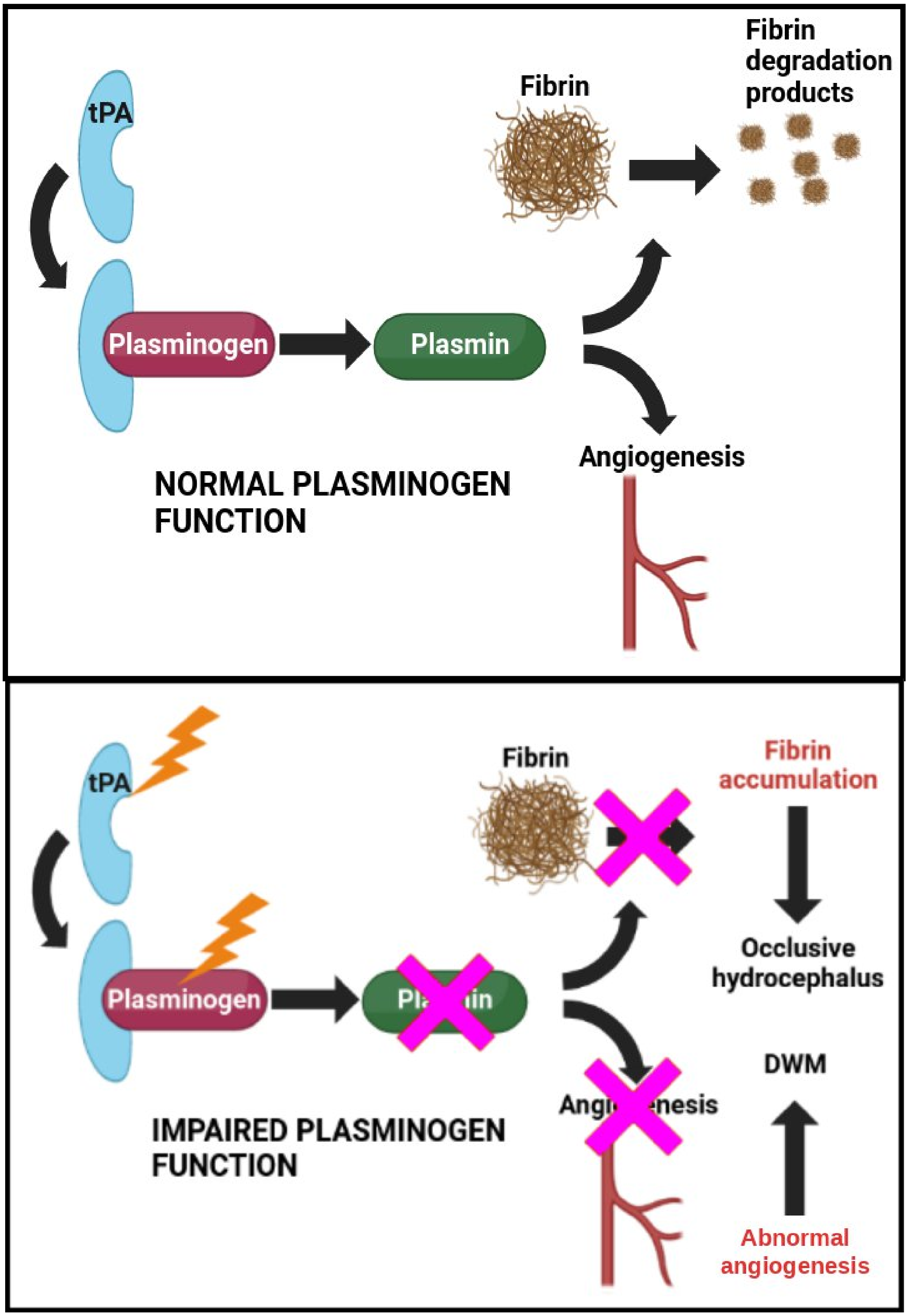

tPA
Plasminogen
Plasmin
Fibrin
Fibrin degradation products
Angiogenesis
NORMAL PLASMINOGEN FUNCTION
tPA
Plasminogen
Fibrin
Fibrin accumulation
Occlusive hydrocephalus
DWM
Abnormal angiogenesis
IMPAIRED PLASMINOGEN FUNCTION
